# Models and observations agree on fewer and milder midlatitude cold extremes even over recent decades of rapid Arctic warming

**DOI:** 10.1126/sciadv.adp1346

**Published:** 2024-10-02

**Authors:** Russell Blackport, Michael Sigmond, James A. Screen

**Affiliations:** ^1^ Canadian Centre for Climate Modelling and Analysis, Environment and Climate Change Canada, Victoria, BC, Canada.; ^2^ Department of Mathematics and Statistics, University of Exeter, Exeter, UK.

## Abstract

An apparent increase in observed cold extremes over recent decades in the northern midlatitudes has been reported, in contrast to robust decreases predicted by climate models. This discrepancy has led to suggestions that models fail to accurately simulate changes in weather patterns caused by Arctic warming. Here, we show that the observed frequency and intensity of midlatitude cold extremes have strongly decreased since 1990 and are consistent with modeled trends. The previously reported increase in cold extremes was overestimated due to an artifact of changing data coverage. We also show that the fraction of land with observed cold extreme increases over recent decades is consistent with model internal variability on top of a near-uniform forced reduction in cold extremes across the midlatitudes. Our results provide strong evidence of a decrease in midlatitude cold extremes over recent decades and consistency between models and observations.

## Teaser

Midlatitude extreme cold events have decreased in severity and frequency over recent decades, in agreement with models.

## INTRODUCTION

Under the background of ongoing global warming, it has been reported that there has been an increase in frequency and intensity of winter cold extremes over the Northern Hemisphere midlatitudes since 1990 (*1*–*4*). This contrasts with long-term trends (*5*–*7*) and climate model projections (*7*, *8*) that show a very robust decline in the frequency and intensity of cold extremes throughout the midlatitudes in response to increased greenhouse gases. The apparent increase in cold extremes coincided with the emergence of rapid Arctic warming and sea ice loss, motivating investigation into the links between them (*1*–*3*, *9*–*16*). Many studies have since invoked changes in the large-scale atmospheric circulation induced by Arctic warming to explain the apparent increase in cold extremes (*1*–*4*, *9*, *13*, *14*, *17*) and argued that models may poorly represent these changes (*2*, *12*). The apparent discrepancy between models and observations (*2*) leads to lack of confidence in model projections and makes it difficult for policy-makers to prepare for the consequences of our changing climate.

Despite the prominence of the narrative of a model-observation discrepancy in cold extreme trends throughout the literature (*2*–*4*, *9*, *17*), there are several reasons to question whether the discrepancy exists. First, few studies have directly compared observed trends (or, more specifically, trends from observation-based products) to modeled trends in cold extremes over recent decades. Those that do have typically only compared the average of many simulations to observed trends over short time periods (*2*, *4*). However, this is not a like-for-like comparison because internal variability is removed when averaging over many simulations, while internal variability can have a large influence on observed trends, particularly over short time periods (*18*–*21*). A few studies have compared observed trends to the distribution of modeled temperature trends, but only over specific regions where winter cooling trends have occurred in observations (*22*–*25*). These studies have found that observed trends can be reproduced in models, but very rarely. While some have argued this is evidence of a model bias (*15*), selecting the regions with the most extreme observed trends to compare to model trends introduces selection bias (*20*). In addition, studies that have compared models and observations typically only examined winter mean temperature trends, which may differ from trends in extremes because of changes in subseasonal variability (*26*, *27*). Last, many of the short-term trends in midlatitude circulation that were used to motivate the potential discrepancy have since reversed in recent years (*28*, *29*).

Here, we revisit recent observed trends in winter cold extremes and search for evidence of a model-observation discrepancy by directly comparing trends in models and observation-based products. To ensure that the comparison is like-for-like, we use seven initial-condition, large ensemble model experiments with historical forcing. The large ensembles are required to compare observations to the full range of possible trends that can occur because of internal variability (*20*). Contrary to recent claims, we find that there has been a substantial decrease in frequency and intensity of midlatitude cold extremes over recent decades and that these trends agree with models. We then reconcile our results with previous studies by showing that the reported observed increase in cold extremes in one prominent study—Cohen *et al.* (*1*)—was due to changing spatial coverage of data, which resulted in a spurious trend. Last, we search for a discrepancy between models and observations in the regional trends by comparing the spatial distribution of observed trends in cold extremes to the range of distributions from the models. We find that the observed spatial distribution, including small regions of weak increases in cold extremes, are consistent with internal variability found in individual model simulations.

## RESULTS

### Cold extreme trends in observations and models

We begin by examining the time evolution of the intensity and frequency of observed midlatitude winter cold extremes over recent decades. To represent observations, we primarily use ERA5 (*30*) reanalysis, which has been shown to perform well in capturing temperature extremes (*31*–*33*). Reanalysis products have the advantage of having complete spatial and temporal coverage that is physically consistent. We note, however, that reanalyses are not true observations and may contain artifacts and inhomogeneities. For this reason, we also repeated some of our analysis using the Japanese reanalysis (JRA55) (*34*) and Berkeley Earth station-based gridded observations (*35*). To capture the intensity of cold extremes, we use the lowest daily-averaged near-surface temperature observed in each winter (hereafter TMn) at each grid point. To capture the frequency of cold extremes, we calculate the number of days where the temperature is below the fifth percentile in each winter (hereafter TM5p; see Materials and Methods). Our analysis focuses on trends since 1990 because this is when the increase in cold extremes associated with rapid Arctic warming is often claimed to have begun (*1*). We also examine data going back to 1971 for the longer-term context.

**Fig. 1.**
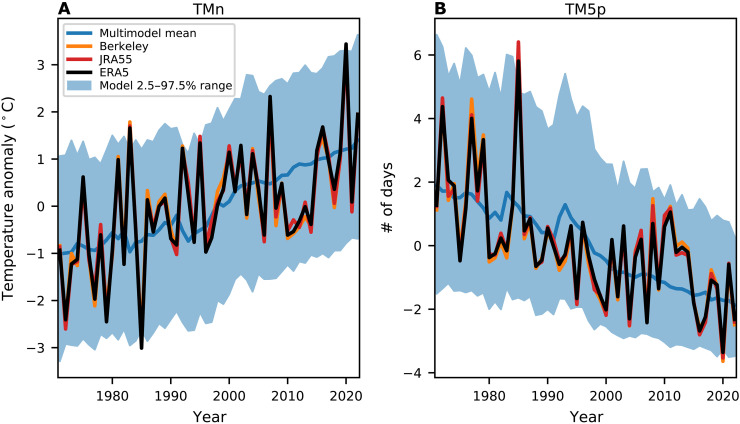
Time evolution of midlatitude cold extremes in models and observations. Time series of anomalies in midlatitude (30 to 60°N, land only) averaged TMn (**A**) and TM5p (**B**) in ERA5 (black), Berkeley Earth (orange), JRA55 (red), and the multimodel mean (blue). The blue shading indicates the 2.5 to 97.5 percentile range of the model spread from all ensemble members. The anomalies are relative to the 1971 to 2022 period.

The time series for ERA5 TMn averaged over the Northern Hemisphere midlatitudes (30 to 60°N, land only) shows a strong increase since 1971, representing a decrease in intensity of cold extremes (Fig. 1A, black line). The linear trend over 1990 to 2022 is 0.42°C per decade, which is similar to the longer-term trend of 0.46°C per decade over 1971 to 2022. The trend of TMn over 1990 to 2022 is statistically significant (*P* ~ 0.02) and is stronger than the global, annual mean trend (0.22°C per decade) and the midlatitude winter mean trend (0.25°C per decade). This amplified warming of extreme cold temperatures relative to the mean is consistent with the observed reduction in subseasonal variability caused by Arctic warming (*26*, *27*, *36*). The time series of ERA5 TM5p is consistent with TMn, displaying a strong reduction in the frequency of cold extremes on top of interannual and decadal variability (Fig. 1B). The trend of TM5p over 1990 to 2022 (−0.47 days per decade) is statistically significant (*P* ~ 0.03) and only slightly weaker than the longer-term trend over 1971 to 2022 (−0.75 days per decade). The time series of TMn and TM5p from JRA55 (Fig. 1, red line) and Berkeley Earth (Fig. 1, orange line) are nearly identical to ERA5, suggesting that these results are unlikely to be an artifact of ERA5. For this reason, the remainder of our analysis will focus on ERA5 reanalysis.

We next compare the ERA5 time series and trends to the model simulations forced with historical forcing. We use data from seven single-model, initial-condition large ensembles each with between 30 and 50 realizations that only differ in their initial conditions, resulting in a total of 300 realizations (see table S1 and Materials and Methods). An issue that arises is that a subset of the latest generation of climate models, including some of the models used here, have very high climate sensitivity and show too much global warming over recent decades relative to observations (*37*–*39*). The purpose of this study is not to evaluate the model climate sensitivities, but instead to evaluate how midlatitude cold extremes change in a warming climate. To account for these known biases in global warming trends, we calculate rescaled model time series and trends based on the ratio of annual mean global warming between the model and observations (see Materials and Methods). Figure 1 shows that the ERA5 time series of both TMn and TM5p metrics are well within the ensemble spread of the rescaled model time series (indicated by the blue shading) with only a few data points outside the 2.5 to 97.5% range. Similarly, when the cold extreme metrics are plotted against annual mean global temperature instead of time, the ERA5 values fall well within the modeled distributions (fig. S1).

**Fig. 2.**
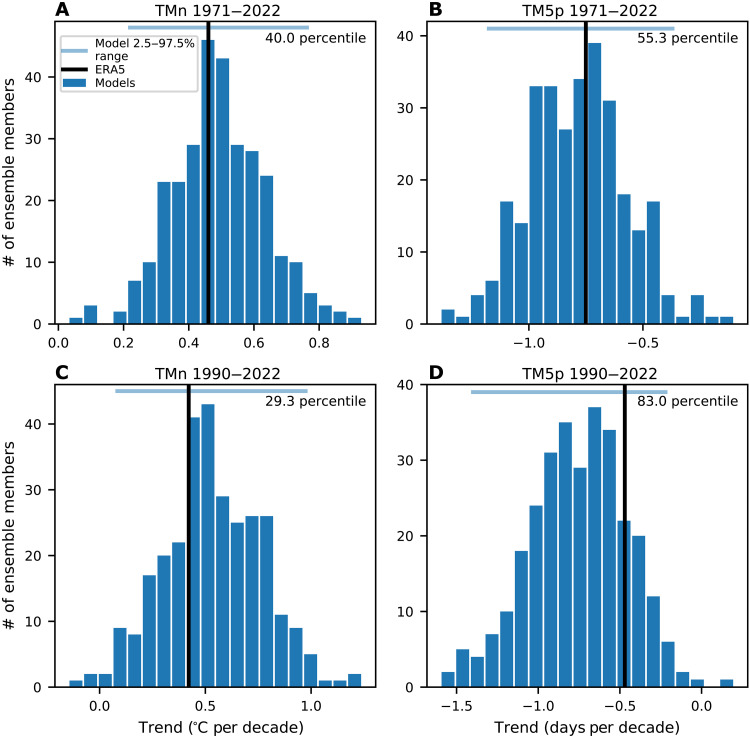
Comparison of trends in cold extremes between models and observations. Histogram of model trends in midlatitude (30 to 60°N, land only) TMn (**A** and **C**) and TM5p (**B** and **D**) over 1971 to 2022 [(A), (B)] and 1990 to 2022 [(C), (D)]. The black vertical line indicates the magnitude of trend in ERA5, and the percentile value of where the ERA5 trend is within the model distribution is indicated in the top right of each panel. The light blue horizontal line indicates the 2.5 to 97.5% model range.

The magnitude of the ERA5 trends over 1971 to 2022 are nearly identical to the multimodel mean and sits in the middle of the ensemble spread (Fig. 2, A and B). For trends over 1990 to 2022, the ERA5 trends are weaker than the ensemble means, but well within the ensemble spread of the models, with 29 and 17% of the realizations showing weaker trends for TMn and TM5p, respectively (Fig. 2, C and D).

Shorter periods such as 1990 to 2013 or 2000 to 2013 are often used (*1*, *2*) to illustrate an increase in extremes and discrepancy between models and observations/reanalysis. In fig. S2, we show a comparison of running 24-year and 14-year trends across all start and end dates between 1971 and 2022. For 24-year running trends, the period around 1990 to 2013 had trends close to zero in ERA5 and were on the very extreme end of the model distribution. However, the most recent periods have seen the ERA5 trends converge toward the model ensemble mean (fig. S2 and Fig. 2, C and D). In addition, the 24-year trends toward the beginning of the period (trends ending in the 1990s and early 2000s) showed stronger reductions in cold extremes relative to the multimodel means. Similarly, the running 14-year trends show that increases in cold extremes for periods ending around 2013 were also unusual, but within the ensemble spread. However, the most recent trends are similarly unusual, but on the opposite side of the distribution (fig. S2). This strongly suggests that internal variability played a large role in the short-term trends from 1990 to 2013 and 2000 to 2013 and shows how sensitive the short-term trends can be to the chosen start and end dates. It is also important to note that the early 1990s was toward the end of a period with a stronger reduction of cold extremes compared to the model mean (fig. S2). The subsequent lack of warming in cold extremes from the 1990s resulted in the convergence of the ERA5 trends that started in 1971 with models (fig. S3). Thus, the usually warm temperatures and lack of cold extremes during the early 1990s likely contributed to the weaker trends starting from about 1990.

The agreement between modeled and ERA5 trends is not sensitive to various choices made in the analysis. Our analysis has focused on comparisons to rescaled modeled data, but the 1990 to 2022 ERA5 trends are still within the model spread if we instead use the model data without any rescaling, but they are further toward the extreme ends of the distribution (fig. S4). If instead of rescaling, we screen for only the models with global warming trends similar to the observations, we see nearly identical results to the rescaled model data (fig. S4). Results are also similar if the daily minimum temperature is used instead of the daily average or if we use the number of days below the 10th percentile instead of the 5th (fig. S4). We also find similar results if a different base period is used to define the percentiles or if we use different definitions of the midlatitudes, such as 30 to 50°N (fig. S4). In addition, the minimum 5- and 9-day running mean temperatures are nearly identical to the TMn data (fig. S4), indicating that persistent cold extremes have substantially decreased in severity, in agreement with model trends.

### Reconciling with previously reported trends

We have shown so far that there was a lack of trends in cold extremes from 1990 to about 2013 (Fig. 1 and fig. S2), despite global warming, but there was not an increase in cold extremes as is sometimes claimed (*1*–*3*). Examining the literature, many of the studies cited to evidence an increase in cold extremes did not analyze trends in cold extremes explicitly (*2*, *3*, *15*), or only examined even shorter periods (<15 years) (*16*, *40*) when internal variability plays an even larger role. One exception, and where the claim that cold extremes have increased appears to originate, is Cohen *et al.* (*1*). They showed a substantial reversal of the long-term trends and a clear increase in cold extremes from the 1990s until 2013 using station-based gridded data from GHCNDEX (*41*).

**Fig. 3.**
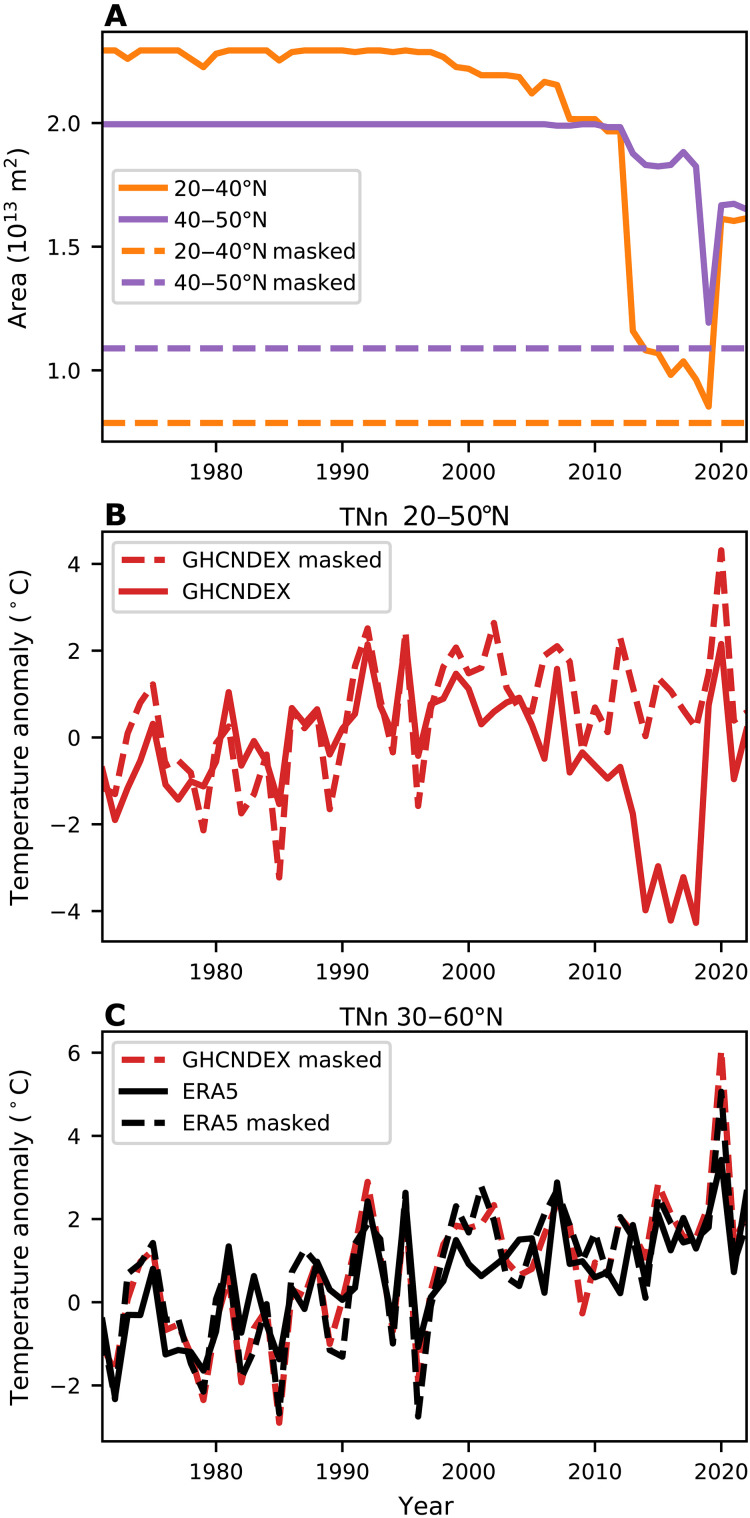
Correcting for artifacts of changing spatial coverage of data. (**A**) Time series of the land area with TNn data from GHCNDEX over 20 to 40°N (orange solid) and 40 to 50°N (purple solid). The dashed lines indicate the land area covered after the mask is applied in each latitude band. (**B**) Time series of TNn averaged over 20 to 50°N from GHCNDEX for the raw data (red solid) and after applying a fixed spatial mask (red dashed). (**C**) Time series of TNn averaged over 30 to 60°N for the masked GHCNDEX (red dashed), ERA5 with no mask (black solid), and ERA5 with the fixed mask from GHCNDEX (black dashed).

A known issue with the GHCNDEX dataset used in Cohen *et al.* (*1*) is that it has spatial coverage that has changed over time (*41*). We illustrate this changing spatial coverage in Fig. 3A, which shows the total land area with data coverage for the coldest daily minimum temperature (TNn) used in Cohen *et al.* (*1*). We break the midlatitude region used by Cohen *et al.* (20 to 50°N) into lower latitudes (20 to 40°N) and higher latitudes (40 to 50°N). Starting around the mid-1990s, there was a decrease in spatial coverage that occurred primarily in the lower latitude (and thus warmer) parts of the midlatitudes (Fig. 3A and fig. S5). Because of changes in spatial coverage like this, it is recommended that a spatial mask is used that accounts for temporal completeness on a per-grid box basis when examining time series and trends of area averaged from GHCNDEX (*41*).

In Fig. 3B, we show the consequences of not accounting for the changing spatial coverage. The raw, unmasked time series shows a remarkable decrease in TNn from the 1990s to the 2010s [indicating more intense cold extremes, as in Cohen *et al.* (*1*)], but also a strong reversal in the most recent years. However, after applying a mask (Fig. 3A and fig. S5) such that only grid points that have complete data over the entire 1971 to 2022 period are used, the decrease in temperature disappears (Fig. 3B) and becomes consistent with the ERA5 time series presented in Fig. 1. Cohen *et al.* (*1*) do not appear to have applied any masking to account for this changing spatial coverage. The lack of masking results in an artificial cooling trend because of the decrease in spatial coverage at lower, warmer latitudes (Fig. 3A).

To directly compare the GHCNDEX data with ERA5 data, we plot the masked GHCNDEX TNn time series together with the TNn time series from ERA5 using the GHCNDEX mask and with no mask (i.e., with complete spatial coverage), averaging over 30 to 60°N (Fig. 3C). The time series and trends for these three datasets are similar to each other, with 1990 to 2022 trends of 0.55°, 0.54°, and 0.44°C per decade, respectively. These are also similar for the TMn metric we use here (1990 to 2022 trends of 0.42°C per decade). Therefore, the differences cannot be explained by different datasets or metrics used. Data coverage issues also exaggerated the trend in icing days also shown in Cohen *et al.* (*1*) (fig. S6). Overall, these results strongly suggest that the increase in cold extremes identified by Cohen *et al.* (*1*) was an artifact of the changing data coverage.

### Spatial distribution of trends

**Fig. 4.**
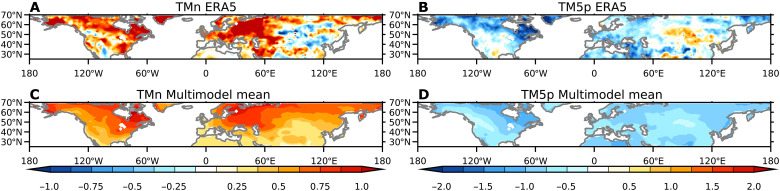
Spatial patterns of trends in cold extremes over 1990 to 2022 in observations and models. Spatial patterns of trends in TMn (**A** and **C**) and TM5p (**B** and **D**) for ERA5 [(A) and (B)] and the multimodel mean [(C) and (D)]. The units of the TMn trends are °C per decade and the units for TM5p trends are days per decade.

The results presented so far show that averaged over the midlatitudes, cold extremes have substantially decreased in recent decades in observations and reanalysis, in agreement with models. However, previous analysis has shown substantial regional variation in the reanalysis trends, with some regions showing increases in cold extremes (*42*). The spatial patterns of the ERA5 trends over 1990 to 2022 show decreases in cold extremes over North America, Europe, and Western Asia, but weak increases over parts of Central and Eastern Asia (Fig. 4, A and B). This is in contrast to the multimodel mean trends that depict a more uniform reduction in cold extremes (Fig. 4, C and D). We now explore this in more detail.

**Fig. 5.**
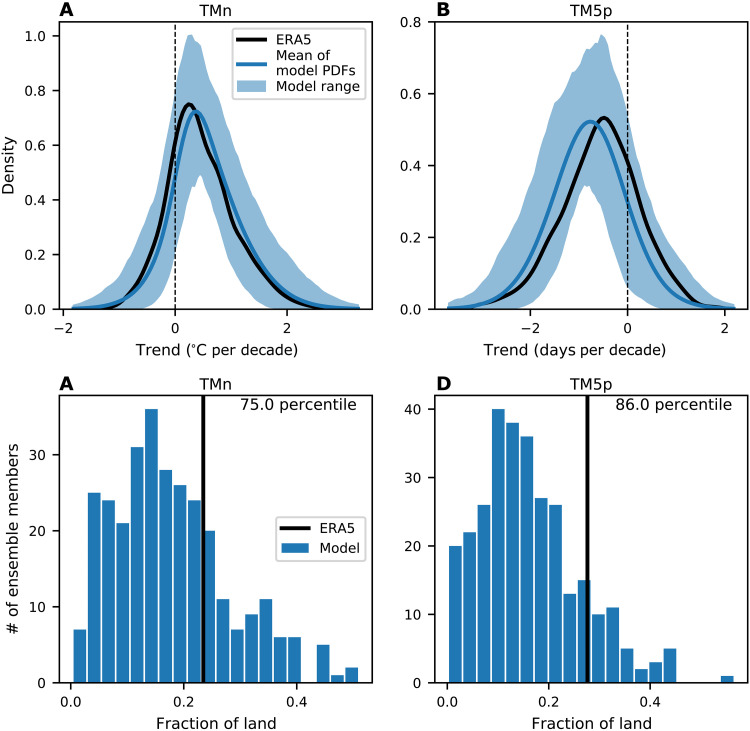
Spatial distribution of cold extreme trends in observations and models. (**A** and **B**) The PDF of the 1990 to 2022 trends at each grid point in the midlatitudes for TMn (A) and TM5p (B). The black line is the PDF for ERA5 trends, the blue line is the multimodel mean of the PDFs from the individual realizations, and the blue shading represents the 2.5 to 97.5% range of the model PDFs. (**C** and **D**) Histograms of the fraction of midlatitude land showing negative 1990 to 2022 trends in TMn (C) and positive trends in TM5p (D). The black vertical line indicates the fraction of land in the ERA5 trends and the percentile value of where the ERA5 trend is within the model distribution is indicated in the top right of each panel.

The regions of weak increases in extremes since 1990 motivate us to examine whether this is different to what would be expected from internal variability in the models. To learn more, we construct a probability density function (PDF) of 1990 to 2022 trends from ERA5 aggregated over all midlatitude land grid points (*43*). Figure 5 (A and B) compares the ERA5 PDF to the range of PDFs calculated from each realization from the models. For both TMn and TM5p, the ERA5 PDFs are well within the model spread and are similar to the ensemble mean of all distributions. The PDFs indicate that all model realizations show some regions where there is an increase in cold extremes. In individual realizations, on average, 18 and 16% of midlatitude land have an increase in extreme cold for TMn and TM5p, respectively. The corresponding values in ERA5 trends are 23 and 27%, which are well within the model spread (Fig. 5, C and D). The individual model realizations indicate that increases in cold extremes can occur anywhere in the midlatitudes (fig. S7). They also suggest that the regions where increases in cold extremes are found in ERA5 coincide with where the models suggest they are most likely to occur (fig. S7). For the longer-term trends since 1971, the PDFs of trends are narrower, but there is better agreement with the ERA5 trends in the middle of the model range (fig. S8). For shorter-term trends since 2000, while the PDFs show a wider range of trends, there is still a strong agreement between models and ERA5 trends (fig. S9).

Previous work has questioned the warming of midlatitude cold extremes because of the lack of statistically significant trends over selected regions in recent ERA5 trends (*42*). However, despite extremely statistically significant (*P* < 10^−12^) ensemble mean trends in the same selected regions, most individual ensemble members show no statistically significant decrease in cold extremes (table S2) because internal variability dominates over the forced response. Thus, it is misleading to interpret the absence of an expected warming trend in small regions as evidence of a compensating forced cooling response to Arctic warming.

## DISCUSSION

We have shown that cold extremes over the northern midlatitudes have decreased in intensity and frequency in observations and reanalysis over the recent period of rapid Arctic warming, in agreement with model simulations. The apparent increase that was previously reported in one prominent study (*1*) was largely an artifact of changing data coverage. After an appropriate fixed mask to account for the changing spatial coverage is applied, or if data with full spatial coverage are used, the increases in cold extremes over the midlatitudes disappear. There was still a period between about 1990 to the early 2010s where trends in cold extremes leveled off, but this was within what is expected from internal variability and occurred following a period of strong decreases in cold extremes. Since then, there has been a decrease in cold extremes and observed trends have now converged with the modeled trends, indicating that the temporary lack of warming was likely a result of internal variability. These results highlight the caution required when interpreting short-term trends that differ from long-term trends and/or models because the discrepancies may not necessarily be due to model error.

Previous studies have used regional cooling or lack of detectable warming seen in recent observations and reanalysis as evidence of model-observation discrepancy and of a regional cooling response to sea ice loss or Arctic warming (*15*, *16*, *42*). However, because of internal variability, it is expected that some regions will warm faster, and others will warm slower than predicted. While short-term trends in reanalysis show some regions with an increase in cold extremes, the percentage of midlatitude land with increases and the spatial distribution of trends are consistent with expectations from model internal variability on top of a near-uniform decrease in cold extremes. We find that despite a near spatially uniform decrease in cold extremes over the entire midlatitudes when averaging over many ensemble members and models, every single model realization shows regions with weak increases in cold extremes from 1990 to 2022 that arise entirely from internal variability. In other words, over short time periods, regional increases in cold extremes arising from internal variability are not only possible; they are virtually guaranteed to occur. Thus, highlighting regions with weak increases or nonsignificant trends (*42*) in cold extremes as evidence of a forced response to Arctic amplification or of a model-observation discrepancy is not appropriate.

The apparent and unexpected increase in cold extremes in recent decades and the discrepancies between model and observation-based studies continues to motivate a large number of studies to examine the cause, with much of the focus on the response to sea ice loss or Arctic warming (*2*, *3*, *44*). Although it is impossible to completely rule out model errors in the forced response to Arctic warming, the lack of evidence we find for an increase in cold extremes or of a model-observation discrepancy suggests that many of these studies are attempting to solve a problem that does not exist.

The decrease in cold extremes is consistent with the weak and nonrobust observed trends in the winter atmospheric circulation variability, which are also in agreement with model circulation trends (*29*). The agreement between models and observations increases confidence in the model projections of substantial decreases in the frequency and intensity of cold extremes over the midlatitudes as the world continues to warm in response to rising greenhouse gas concentrations. We caution, however, that because of internal variability, temporary, regional increases in cold extremes are still expected to occur.

## MATERIALS AND METHODS

### Observations and reanalysis

Most of our analysis uses daily average near-surface temperature from ERA5 (*30*) reanalysis over 1970 to 2022. The hourly data were downloaded on the native 0.25° × 0.25° latitude-longitude grid before taking the daily average and bilinearly interpolating to a 2° × 2° latitude-longitude grid. We define winter to be December-January-February, where the labeled year is the year that corresponds to the January-February year (e.g., 2022 refers to December 2021, January 2022, February 2022). The longer-term trends are chosen to start in 1971 because one of the models used (EC-Earth3) only has data available back to 1970. In addition, it is around this time that the recent near-linear trend in global warming began. Our conclusions about the longer-term trends are not sensitive to changes in this start year. We also use daily averaged near–surface temperature data from JRA-55 (*34*) and Berkeley Earth (*35*) and the gridded annual minimum temperature (TNn) and icing days data from GHCNDEX (*41*). For the JRA-55 data, the 6-hourly data on the 1.25° grid was downloaded before taking the daily average and bilinearly interpolated to the 2° × 2° latitude-longitude grid. For Berkeley Earth, the daily average data on the 1° × 1° grid was downloaded and bilinearly interpolated to the 2° × 2° latitude-longitude grid. The analysis of the GHCNDEX was done on the native grid without any interpolation.

### Model experiments

We use daily average near surface temperature from the initial-condition large ensemble from seven different models (table S1). These are ACCESS-ESM1-5 (*45*), CanESM5 (*46*), CESM2 (*47*, *48*), EC-Earth3 (*49*), GFDL-SPEAR-MED (*50*), MIROC6 (*51*, *52*), and MPI-ESM1-2-LR (*53*). These are all the models (to our knowledge) from the latest generation of models that have large ensembles with at least 30 members over the 1971 to 2022 period at the time the analysis was performed. For the CESM2 model, a total of 100 ensemble members exist, but 50 of the members used historical biomass burning forcing that is different to that of Coupled Model Intercomparison Project Phase 6 (CMIP6). To be consistent with the other models we have only analyzed the 50 members that include the forcing from CMIP6. The different ensemble members within each model ensemble differ only in their initial conditions. All models are forced with historical forcing until 2014 and then projected forcing thereafter. The projected forcing used is different for each model, depending on data availability (table S1). The different scenarios are unlikely to have a large effect on the results because the projected forcings are similar over the period we have analyzed (2015 to 2022), and our rescaling will account for minor differences in global mean temperatures.

All data were bilinearly interpolated to a common 2° × 2° latitude-longitude grid before the analysis was performed. For the multimodel means, averages of each model are calculated first, before averaging over all models for the multimodel mean. For the analysis of the ensemble spread, all 300 realizations are pooled together, and each realization is weighted equally.

### Metrics

The TMn metric is the minimum daily average temperature in each winter. This is similar to the TNn metric that is commonly used (*54*), but with daily averaged temperature instead of daily minimum temperatures, and only considering the winter months instead of all months. We chose to use TMn instead of TNn because we are interested in changes on daily and longer timescales potentially associated with Arctic warming. The TNn metric includes changes in the diurnal cycle, which is not the focus of this study. Extreme indices that use daily averaged temperature instead of minimum temperature have been previously used to study the potential links between the Arctic and midlatitudes (*42*, *55*). To calculate TNn from ERA5, we calculate the minimum temperature from the instantaneous hourly data. For fig. S4, we calculate the minimum temperature over winter, but for Fig. 3C, we calculate the minimum temperature over the calendar year to be consistent with the GHCNDEX data.

TM5p is the number of winter days where the daily average temperature is below the fifth percentile. The fifth percentile is based on a daily climatology using a rolling window of 5 days centered on the day of interest. We use the entire period (1971 to 2022) for the climatological base period to avoid the inhomogeneities associated with transitioning from within to outside the base period (*56*). While the magnitude of the trends can be sensitive to the choice of base period (*57*), we find that the differences are similar between models and reanalysis. This means that where the reanalysis trends sit within the model distributions (fig. S4) and our conclusions are not sensitive to the choice of base period. In the models, the climatological values are calculated in each individual realization, not on the whole ensemble, to make the comparison to reanalysis like-for-like. Statistical significance of the trends is evaluated using a two-tailed Student’s *t* test with a significance threshold of *P* = 0.05.

### Spatial PDFs

The spatial PDFs in Fig. 5 are calculated by first taking the trends at each land grid point between 30 and 60°N. We then estimate the PDFs of the spatial distribution with a Gaussian kernel-density estimator using “gaussian_kde” from the python (v3.8.3) module scipy.stats (v1.5.0). The “scott” method is used to calculate the estimator bandwidth. The grid points are appropriately weighted by the area in the PDF estimation.

### Model rescaling

To account for the differences in the magnitude of global warming between models and observations, we apply a rescaling to the model trends and time series. A simple approach would be to multiply the modeled trend in the cold extremes in each ensemble member by the ratio of observed to modelled global warming trend. This is equivalent to calculating the change in cold extreme metric per degree of global warming. This would be an adequate approach if the ensemble mean was the only quantity of interest. However, this method artificially alters the ensemble spread, which is not desirable. This is because the signal-to-noise ratio remains constant when dividing each member by the global warming ratio; thus, while the signal is rescaled, so is the noise. This would then assume that the magnitude of internal variability increases with the magnitude of global warming, such that a doubling of global warming leads to a doubling of the magnitude of internal variability. This assumption is false, as climate models show either no change or a decrease in the magnitude of internal variability with global warming, as discussed below.

To properly rescale the model trends without altering the ensemble spread, we apply the following rescaling

$$R_{m,i}=B_{m,i}+E_{m}\left(1-\frac{G_{m}}{G_{o}}\right)$$

where *R*_*m*, *i*_ is the rescaled trend in ensemble member *i* in the model *m*, *B*_*m*, *i*_ is the corresponding trend in the raw model data before rescaling, *E_m_* is the ensemble mean trend in the model, *G_m_* is the global, annual mean temperature trend in the model, and *G_o_* is the observed trend in global, annual mean temperature from ERA5. This calculates the magnitude the ensemble mean needs to be rescaled by according to the ratio of global warming and then shifts the entire distribution by this amount without changing the shape of this distribution. The global warming ratio is calculated using the global mean temperature trend over the period that is being examined.

This rescaling approach assumes that internal variability on the timescales we examined here remains approximately constant under global warming levels seen in the observations and models over the historical period. It also assumes that the metrics used change linearly with global warming. These assumptions are likely to be approximately true for TMn (Fig. 1A and fig. S1), but TM5p does appear to show some decrease in variability and nonlinearity with warming (Fig. 1B and fig. S1). Both of these are likely because the TM5p metric is bounded by zero. The decrease in variability implies that models with too much global warming likely show too little spread in the rescaled TM5p. The nonlinearity implies that models that show too much global warming will have weaker rescaled trends than they should. Thus, the model spread for TM5p in our analysis may be underestimated, but the trends may be too small. For ERA5 trends that are weaker than the ensemble mean (e.g., Fig. 2D), these two effects compete against each other for where the ERA5 trends fits within the model distribution, but the exact magnitude of each is not clear.

This procedure is applied after spatial averaging of the metrics for the analysis of the midlatitude average and at each grid point for the analysis of the spatial maps and distributions. The time series have been rescaled the same way with the appropriate linear trend removed. The results are nearly identical if the midlatitude land are excluded from the global mean calculation.

In addition to the rescaling, we also apply a screening criterion in fig. S4 where we select only the models with global warming similar to observations. The criterion we use is whether the model global warming trend is within 20% of the observed global warming trend. This results in two models being included in the analysis: MIROC6 and MPI-ESM1-2-LR.

## References

[1] J. Cohen, J. A. Screen, J. C. Furtado, M. Barlow, D. Whittleston, D. Coumou, J. Francis, K. Dethloff, D. Entekhabi, J. Overland, J. Jones, Recent Arctic amplification and extreme mid-latitude weather. Nat. Geosci. 7, 627–637 (2014).

[2] J. Cohen, X. Zhang, J. Francis, T. Jung, R. Kwok, J. Overland, T. J. Ballinger, U. S. Bhatt, H. W. Chen, D. Coumou, S. Feldstein, H. Gu, D. Handorf, G. Henderson, M. Ionita, M. Kretschmer, F. Laliberte, S. Lee, H. W. Linderholm, W. Maslowski, Y. Peings, K. Pfeiffer, I. Rigor, T. Semmler, J. Stroeve, P. C. Taylor, S. Vavrus, T. Vihma, S. Wang, M. Wendisch, Y. Wu, J. Yoon, Divergent consensuses on Arctic amplification influence on midlatitude severe winter weather. Nat. Clim. Chang. 10, 20–29 (2020).

[3] J. Cohen, L. Agel, M. Barlow, C. I. Garfinkel, I. White, Linking Arctic variability and change with extreme winter weather in the United States. Science 373, 1116–1121 (2021). 34516838 10.1126/science.abi9167

[4] J. L. Cohen, J. C. Furtado, M. A. Barlow, V. A. Alexeev, J. E. Cherry, Arctic warming, increasing snow cover and widespread boreal winter cooling. Environ. Res. Lett. 7, 014007 (2012).

[5] G. J. van Oldenborgh, E. Mitchell-Larson, G. A. Vecchi, H. de Vries, R. Vautard, F. Otto, Cold waves are getting milder in the northern midlatitudes. Environ. Res. Lett. 14, 114004 (2019).

[6] R. J. H. Dunn, L. V. Alexander, M. G. Donat, X. Zhang, M. Bador, N. Herold, T. Lippmann, R. Allan, E. Aguilar, A. A. Barry, M. Brunet, J. Caesar, G. Chagnaud, V. Cheng, T. Cinco, I. Durre, R. de Guzman, T. M. Htay, W. M. W. Ibadullah, M. K. I. B. Ibrahim, M. Khoshkam, A. Kruger, H. Kubota, T. W. Leng, G. Lim, L. Li-Sha, J. Marengo, S. Mbatha, S. McGree, M. Menne, M. de los Milagros Skansi, S. Ngwenya, F. Nkrumah, C. Oonariya, J. D. Pabon-Caicedo, G. Panthou, C. Pham, F. Rahimzadeh, A. Ramos, E. Salgado, J. Salinger, Y. Sané, A. Sopaheluwakan, A. Srivastava, Y. Sun, B. Timbal, N. Trachow, B. Trewin, G. van der Schrier, J. Vazquez-Aguirre, R. Vasquez, C. Villarroel, L. Vincent, T. Vischel, R. Vose, M. N. A. B. H. Yussof, Development of an updated global land in situ-based data set of temperature and precipitation extremes: HadEX3. J. Geophys. Res. 125, e2019JD032263 (2020).

[7] S. I. Seneviratne, X. Zhang, M. Adnan, W. Badi, C. Dereczynski, A. D. Luca, S. Ghosh, I. Iskandar, J. Kossin, S. Lewis, F. Otto, I. Pinto, M. Satoh, S. M. Vicente-Serrano, M. Wehner, B. Zhou, Weather and climate extreme events in a changing climate, in *Climate Change 2021—The Physical Science Basis: Working Group I Contribution to the Sixth Assessment Report of the Intergovernmental Panel on Climate Change* (Cambridge Univ. Press, ed. 1, 2021), pp. 1513–1766.

[8] C. Li, F. Zwiers, X. Zhang, G. Li, Y. Sun, M. Wehner, Changes in annual extremes of daily temperature and precipitation in CMIP6 models. J. Climate 34, 3441–3460 (2021).

[9] J. Cohen, K. Pfeiffer, J. A. Francis, Warm Arctic episodes linked with increased frequency of extreme winter weather in the United States. Nat. Commun. 9, 869 (2018). 29535297 10.1038/s41467-018-02992-9 PMC5849726

[10] J.-S. Kug, J.-H. Jeong, Y.-S. Jang, B.-M. Kim, C. K. Folland, S.-K. Min, S.-W. Son, Two distinct influences of Arctic warming on cold winters over North America and East Asia. Nat. Geosci. 8, 759–762 (2015).

[11] M. Mori, M. Watanabe, H. Shiogama, J. Inoue, M. Kimoto, Robust Arctic sea-ice influence on the frequent Eurasian cold winters in past decades. Nat. Geosci. 7, 869–873 (2014).

[12] M. Mori, Y. Kosaka, M. Watanabe, H. Nakamura, M. Kimoto, A reconciled estimate of the influence of Arctic sea-ice loss on recent Eurasian cooling. Nat. Clim. Chang. 9, 123–129 (2019).

[13] J. A. Francis, S. J. Vavrus, Evidence linking Arctic amplification to extreme weather in mid-latitudes. Geophys. Res. Lett. 39, L06801 (2012).

[14] J. A. Francis, S. J. Vavrus, Evidence for a wavier jet stream in response to rapid Arctic warming. Environ. Res. Lett. 10, 014005 (2015).

[15] J. E. Overland, T. J. Ballinger, J. Cohen, J. A. Francis, E. Hanna, R. Jaiser, B.-M. Kim, S.-J. Kim, J. Ukita, T. Vihma, M. Wang, X. Zhang, How do intermittency and simultaneous processes obfuscate the Arctic influence on midlatitude winter extreme weather events? Environ. Res. Lett. 16, 043002 (2021).

[16] J. Overland, J. A. Francis, R. Hall, E. Hanna, S.-J. Kim, T. Vihma, The melting Arctic and Midlatitude weather patterns: Are they connected? J. Climate 28, 7917–7932 (2015).

[17] J. Cohen, L. Agel, M. Barlow, C. I. Garfinkel, I. White, Arctic change reduces risk of cold extremes—Response. Science 375, 729–730 (2022). 10.1126/science.abn8954 35175816

[18] T. G. Shepherd, Atmospheric circulation as a source of uncertainty in climate change projections. Nat. Geosci. 7, 703–708 (2014).

[19] C. Deser, F. Lehner, K. B. Rodgers, T. Ault, T. L. Delworth, P. N. DiNezio, A. Fiore, C. Frankignoul, J. C. Fyfe, D. E. Horton, J. E. Kay, R. Knutti, N. S. Lovenduski, J. Marotzke, K. A. McKinnon, S. Minobe, J. Randerson, J. A. Screen, I. R. Simpson, M. Ting, Insights from Earth system model initial-condition large ensembles and future prospects. Nat. Clim. Chang. 10, 277–286 (2020).

[20] S. Jain, A. A. Scaife, T. G. Shepherd, C. Deser, N. Dunstone, G. A. Schmidt, K. E. Trenberth, T. Turkington, Importance of internal variability for climate model assessment. Npj Clim. Atmos. Sci. 6, 1–7 (2023).

[21] F. Lehner, C. Deser, Origin, importance, and predictive limits of internal climate variability. Environ. Res. Clim. 2, 023001 (2023).

[22] K. E. McCusker, J. C. Fyfe, M. Sigmond, Twenty-five winters of unexpected Eurasian cooling unlikely due to Arctic sea-ice loss. Nat. Geosci. 9, 838–842 (2016).

[23] L. Sun, J. Perlwitz, M. Hoerling, What caused the recent “Warm Arctic, Cold Continents” trend pattern in winter temperatures? Geophys. Res. Lett. 43, 5345–5352 (2016).

[24] F. Ogawa, N. Keenlyside, Y. Gao, T. Koenigk, S. Yang, L. Suo, T. Wang, G. Gastineau, T. Nakamura, H. N. Cheung, N.-E. Omrani, J. Ukita, V. Semenov, Evaluating impacts of recent Arctic sea ice loss on the Northern Hemisphere winter climate change. Geophys. Res. Lett. 45, 3255–3263 (2018).

[25] M. Sigmond, J. C. Fyfe, Tropical Pacific impacts on cooling North American winters. Nat. Clim. Chang. 6, 970–974 (2016).

[26] J. A. Screen, Arctic amplification decreases temperature variance in northern mid- to high-latitudes. Nat. Clim. Chang. 4, 577–582 (2014).

[27] R. Blackport, J. C. Fyfe, J. A. Screen, Decreasing subseasonal temperature variability in the northern extratropics attributed to human influence. Nat. Geosci. 14, 719–723 (2021).

[28] R. Blackport, J. A. Screen, Weakened evidence for mid-latitude impacts of Arctic warming. Nat. Clim. Chang. 10, 1065–1066 (2020).

[29] R. Blackport, J. A. Screen, Insignificant effect of Arctic amplification on the amplitude of midlatitude atmospheric waves. Sci. Adv. 6, eaay2880 (2020). 32128402 10.1126/sciadv.aay2880 PMC7030927

[30] H. Hersbach, B. Bell, P. Berrisford, S. Hirahara, A. Horányi, J. Muñoz-Sabater, J. Nicolas, C. Peubey, R. Radu, D. Schepers, A. Simmons, C. Soci, S. Abdalla, X. Abellan, G. Balsamo, P. Bechtold, G. Biavati, J. Bidlot, M. Bonavita, G. De Chiara, P. Dahlgren, D. Dee, M. Diamantakis, R. Dragani, J. Flemming, R. Forbes, M. Fuentes, A. Geer, L. Haimberger, S. Healy, R. J. Hogan, E. Hólm, M. Janisková, S. Keeley, P. Laloyaux, P. Lopez, C. Lupu, G. Radnoti, P. de Rosnay, I. Rozum, F. Vamborg, S. Villaume, J.-N. Thépaut, The ERA5 global reanalysis. Q. J. R. Meteorol. Soc. 146, 1999–2049 (2020).

[31] S. C. Sheridan, C. C. Lee, E. T. Smith, A comparison between station observations and reanalysis data in the identification of extreme temperature events. Geophys. Res. Lett. 47, e2020GL088120 (2020).

[32] J. D. Keller, S. Wahl, Representation of climate in reanalyses: An intercomparison for Europe and North America. J. Climate 34, 1667–1684 (2021).

[33] R. J. H. Dunn, M. G. Donat, L. V. Alexander, Comparing extremes indices in recent observational and reanalysis products. Front. Clim. 4, 10.3389/fclim.2022.989505 (2022).

[34] S. Kobayashi, Y. Ota, Y. Harada, A. Ebita, M. Moriya, H. Onoda, K. Onogi, H. Kamahori, C. Kobayashi, H. Endo, K. Miyaoka, K. Takahashi, The JRA-55 reanalysis: General specifications and basic characteristics. J. Meteorol. Soc. Jpn. Ser. II 93, 5–48 (2015).

[35] R. A. Rohde, Z. Hausfather, The Berkeley Earth land/ocean temperature record. Earth Sys. Sci. Data 12, 3469–3479 (2020).

[36] A. Dai, J. Deng, Arctic amplification weakens the variability of daily temperatures over northern middle-high latitudes. J. Climate 34, 2591–2609 (2021).

[37] Z. Hausfather, K. Marvel, G. A. Schmidt, J. W. Nielsen-Gammon, M. Zelinka, Climate simulations: Recognize the ‘hot model’ problem. Nature 605, 26–29 (2022). 35508771 10.1038/d41586-022-01192-2

[38] K. B. Tokarska, M. B. Stolpe, S. Sippel, E. M. Fischer, C. J. Smith, F. Lehner, R. Knutti, Past warming trend constrains future warming in CMIP6 models. Sci. Adv. 6, eaaz9549 (2020). 32206725 10.1126/sciadv.aaz9549 PMC7080456

[39] Y. Liang, N. P. Gillett, A. H. Monahan, Climate model projections of 21st century global warming constrained using the observed warming trend. Geophys. Res. Lett. 47, e2019GL086757 (2020).

[40] N. C. Johnson, S.-P. Xie, Y. Kosaka, X. Li, Increasing occurrence of cold and warm extremes during the recent global warming slowdown. Nat. Commun. 9, 1724 (2018). 29712890 10.1038/s41467-018-04040-y PMC5928063

[41] M. G. Donat, L. V. Alexander, H. Yang, I. Durre, R. Vose, J. Caesar, Global land-based datasets for monitoring climatic extremes. Bull. Am. Meteorol. Soc. 94, 997–1006 (2013).

[42] J. Cohen, L. Agel, M. Barlow, D. Entekhabi, No detectable trend in mid-latitude cold extremes during the recent period of Arctic amplification. Commun. Earth Environ. 4, 1–9 (2023).37325084

[43] E. M. Fischer, U. Beyerle, R. Knutti, Robust spatially aggregated projections of climate extremes. Nat. Clim. Chang. 3, 1033–1038 (2013).

[44] D. M. Smith, R. Eade, M. B. Andrews, H. Ayres, A. Clark, S. Chripko, C. Deser, N. J. Dunstone, J. García-Serrano, G. Gastineau, L. S. Graff, S. C. Hardiman, B. He, L. Hermanson, T. Jung, J. Knight, X. Levine, G. Magnusdottir, E. Manzini, D. Matei, M. Mori, R. Msadek, P. Ortega, Y. Peings, A. A. Scaife, J. A. Screen, M. Seabrook, T. Semmler, M. Sigmond, J. Streffing, L. Sun, A. Walsh, Robust but weak winter atmospheric circulation response to future Arctic sea ice loss. Nat. Commun. 13, 727 (2022). 35132058 10.1038/s41467-022-28283-y PMC8821642

[45] T. Ziehn, M. A. Chamberlain, R. M. Law, A. Lenton, R. W. Bodman, M. Dix, L. Stevens, Y.-P. Wang, J. Srbinovsky, The Australian Earth System Model: ACCESS-ESM1.5. J. S. Hemis. Earth Syst. Sci. 70, 193–214 (2020).

[46] N. C. Swart, J. N. S. Cole, V. V. Kharin, M. Lazare, J. F. Scinocca, N. P. Gillett, J. Anstey, V. Arora, J. R. Christian, S. Hanna, Y. Jiao, W. G. Lee, F. Majaess, O. A. Saenko, C. Seiler, C. Seinen, A. Shao, M. Sigmond, L. Solheim, K. von Salzen, D. Yang, B. Winter, The Canadian Earth System Model version 5 (CanESM5.0.3). Geosci. Model Dev. 12, 4823–4873 (2019).

[47] G. Danabasoglu, J.-F. Lamarque, J. Bacmeister, D. A. Bailey, A. K. DuVivier, J. Edwards, L. K. Emmons, J. Fasullo, R. Garcia, A. Gettelman, C. Hannay, M. M. Holland, W. G. Large, P. H. Lauritzen, D. M. Lawrence, J. T. M. Lenaerts, K. Lindsay, W. H. Lipscomb, M. J. Mills, R. Neale, K. W. Oleson, B. Otto-Bliesner, A. S. Phillips, W. Sacks, S. Tilmes, L. van Kampenhout, M. Vertenstein, A. Bertini, J. Dennis, C. Deser, C. Fischer, B. Fox-Kemper, J. E. Kay, D. Kinnison, P. J. Kushner, V. E. Larson, M. C. Long, S. Mickelson, J. K. Moore, E. Nienhouse, L. Polvani, P. J. Rasch, W. G. Strand, The Community Earth System Model version 2 (CESM2). J. Adv. Model. Earth Syst. 12, e2019MS001916 (2020).

[48] K. B. Rodgers, S.-S. Lee, N. Rosenbloom, A. Timmermann, G. Danabasoglu, C. Deser, J. Edwards, J.-E. Kim, I. R. Simpson, K. Stein, M. F. Stuecker, R. Yamaguchi, T. Bódai, E.-S. Chung, L. Huang, W. M. Kim, J.-F. Lamarque, D. L. Lombardozzi, W. R. Wieder, S. G. Yeager, Ubiquity of human-induced changes in climate variability. Earth Syst. Dynam. 12, 1393–1411 (2021).

[49] R. Döscher, M. Acosta, A. Alessandri, P. Anthoni, T. Arsouze, T. Bergman, R. Bernardello, S. Boussetta, L.-P. Caron, G. Carver, M. Castrillo, F. Catalano, I. Cvijanovic, P. Davini, E. Dekker, F. J. Doblas-Reyes, D. Docquier, P. Echevarria, U. Fladrich, R. Fuentes-Franco, M. Gröger, J. v. Hardenberg, J. Hieronymus, M. P. Karami, J.-P. Keskinen, T. Koenigk, R. Makkonen, F. Massonnet, M. Ménégoz, P. A. Miller, E. Moreno-Chamarro, L. Nieradzik, T. van Noije, P. Nolan, D. O’Donnell, P. Ollinaho, G. van den Oord, P. Ortega, O. T. Prims, A. Ramos, T. Reerink, C. Rousset, Y. Ruprich-Robert, P. Le Sager, T. Schmith, R. Schrödner, F. Serva, V. Sicardi, M. S. Madsen, B. Smith, T. Tian, E. Tourigny, P. Uotila, M. Vancoppenolle, S. Wang, D. Wårlind, U. Willén, K. Wyser, S. Yang, X. Yepes-Arbós, Q. Zhang, The EC-Earth3 Earth system model for the Coupled Model Intercomparison Project 6. Geosc. Model Dev. 15, 2973–3020 (2022).

[50] T. L. Delworth, W. F. Cooke, A. Adcroft, M. Bushuk, J.-H. Chen, K. A. Dunne, P. Ginoux, R. Gudgel, R. W. Hallberg, L. Harris, M. J. Harrison, N. Johnson, S. B. Kapnick, S.-J. Lin, F. Lu, S. Malyshev, P. C. Milly, H. Murakami, V. Naik, S. Pascale, D. Paynter, A. Rosati, M. D. Schwarzkopf, E. Shevliakova, S. Underwood, A. T. Wittenberg, B. Xiang, X. Yang, F. Zeng, H. Zhang, L. Zhang, M. Zhao, SPEAR: The next generation GFDL modeling system for seasonal to multidecadal prediction and projection. J. Adv. Model. Earth Syst. 12, e2019MS001895 (2020).

[51] H. Tatebe, T. Ogura, T. Nitta, Y. Komuro, K. Ogochi, T. Takemura, K. Sudo, M. Sekiguchi, M. Abe, F. Saito, M. Chikira, S. Watanabe, M. Mori, N. Hirota, Y. Kawatani, T. Mochizuki, K. Yoshimura, K. Takata, R. O’ishi, D. Yamazaki, T. Suzuki, M. Kurogi, T. Kataoka, M. Watanabe, M. Kimoto, Description and basic evaluation of simulated mean state, internal variability, and climate sensitivity in MIROC6. Geosci. Model Dev. 12, 2727–2765 (2019).

[52] H. Shiogama, H. Tatebe, M. Hayashi, M. Abe, M. Arai, H. Koyama, Y. Imada, Y. Kosaka, T. Ogura, M. Watanabe, MIROC6 Large Ensemble (MIROC6-LE): Experimental design and initial analyses. Earth Syst. Dynam. 14, 1107–1124 (2023).

[53] T. Mauritsen, J. Bader, T. Becker, J. Behrens, M. Bittner, R. Brokopf, V. Brovkin, M. Claussen, T. Crueger, M. Esch, I. Fast, S. Fiedler, D. Fläschner, V. Gayler, M. Giorgetta, D. S. Goll, H. Haak, S. Hagemann, C. Hedemann, C. Hohenegger, T. Ilyina, T. Jahns, D. Jimenéz-de-la-Cuesta, J. Jungclaus, T. Kleinen, S. Kloster, D. Kracher, S. Kinne, D. Kleberg, G. Lasslop, L. Kornblueh, J. Marotzke, D. Matei, K. Meraner, U. Mikolajewicz, K. Modali, B. Möbis, W. A. Müller, J. E. M. S. Nabel, C. C. W. Nam, D. Notz, S.-S. Nyawira, H. Paulsen, K. Peters, R. Pincus, H. Pohlmann, J. Pongratz, M. Popp, T. J. Raddatz, S. Rast, R. Redler, C. H. Reick, T. Rohrschneider, V. Schemann, H. Schmidt, R. Schnur, U. Schulzweida, K. D. Six, L. Stein, I. Stemmler, B. Stevens, J.-S. von Storch, F. Tian, A. Voigt, P. Vrese, K.-H. Wieners, S. Wilkenskjeld, A. Winkler, E. Roeckner, Developments in the MPI-M Earth System Model version 1.2 (MPI-ESM1.2) and its response to increasing CO_2_. J. Adv. Model Earth Syst. 11, 998–1038 (2019). 32742553 10.1029/2018MS001400 PMC7386935

[54] X. Zhang, L. Alexander, G. C. Hegerl, P. Jones, A. K. Tank, T. C. Peterson, B. Trewin, F. W. Zwiers, Indices for monitoring changes in extremes based on daily temperature and precipitation data. WIREs Clim. Chang. 2, 851–870 (2011).

[55] J. A. Screen, C. Deser, L. Sun, Reduced risk of North American cold extremes due to continued Arctic sea ice loss. Bull. Am. Meteorol. Soc. 96, 1489–1503 (2015).

[56] X. Zhang, G. Hegerl, F. W. Zwiers, J. Kenyon, Avoiding inhomogeneity in percentile-based indices of temperature extremes. J. Climate 18, 1641–1651 (2005).

[57] R. J. H. Dunn, C. P. Morice, On the effect of reference periods on trends in percentile-based extreme temperature indices. Environ. Res. Lett. 17, 034026 (2022).

