# Supplementary material for: Models and observations agree on fewer and milder midlatitude cold extremes even over recent decades of rapid Arctic warming
Source: Sci Adv. 2024 Oct 2;10(40):eadp1346. doi: 10.1126/sciadv.adp1346 (PMC11446279; doi:10.1126/sciadv.adp1346)
Supplement: Supplementary file 1 — Figs. S1 to S9 Tables S1 and S2 [file sciadv.adp1346_sm.pdf]

Science Advances
AAAS

Supplementary Materials for

Models and observations agree on fewer and milder midlatitude cold extremes even over recent decades of rapid Arctic warming

Russell Blackport *et al.*

Corresponding author: Russell Blackport, russell.blackport@ec.gc.ca

Sci. Adv. **10**, eadp1346 (2024)
DOI: 10.1126/sciadv.adp1346

This PDF file includes:

Figs. S1 to S9
Tables S1 and S2

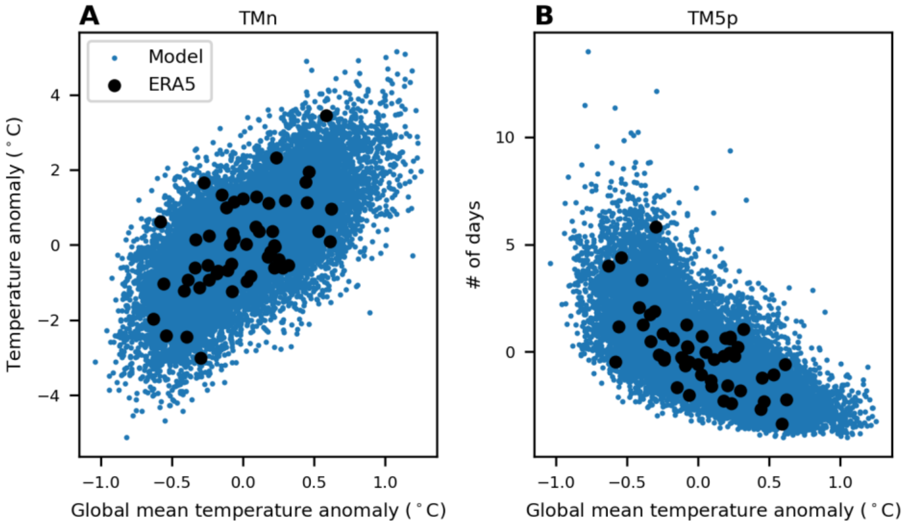

Fig. S1. Scatter plot of cold extremes against the global mean temperature. Scatter plot of the anomaly in midlatitude TMn (**A**) and TM5p (**B**) against the annual global mean temperature anomalies in each model realization (blue) and ERA5 (black) over 1971-2022. No rescaling was done for the model data. Anomalies are calculated relative to the 1971-2022 period.

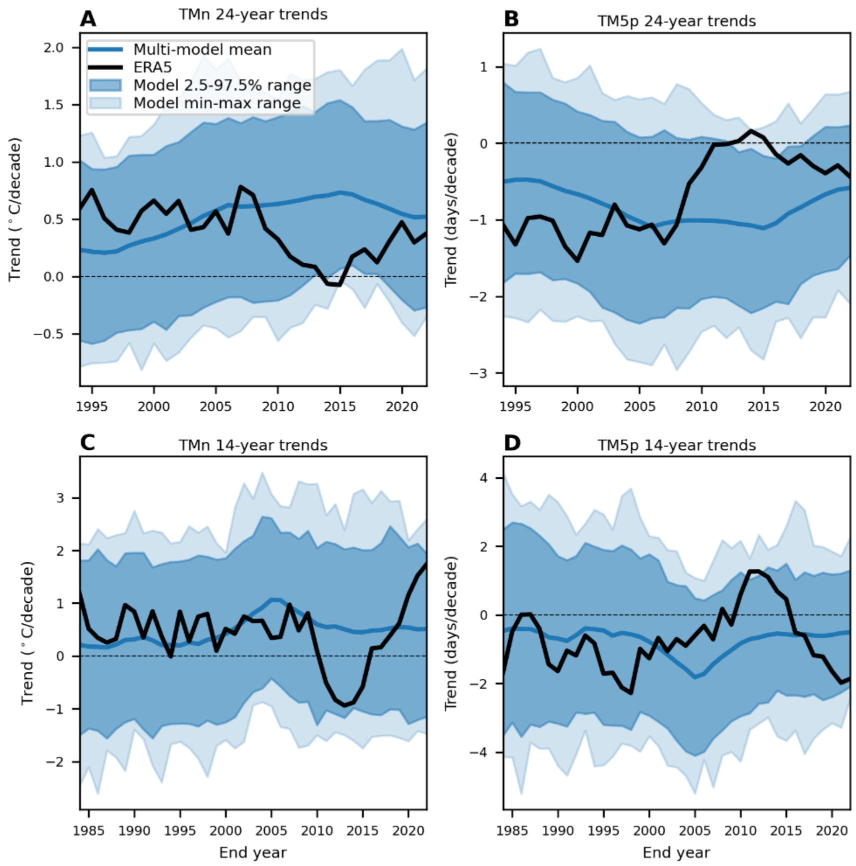

Fig. S2. Trends in models and observations for a fixed length running window. **A, B** The magnitude of the ERA5 trends (black) as function of end year for a 24-year running window for TMn (A)and TM5p (B). The blue line indicates the magnitude of the trends of the multi-model mean, the dark blue shading shows the 2.5-97.5% model range, and the light blue shading shows that full min-max model range. **C**,**D** As in A,B but for 14-year trends.

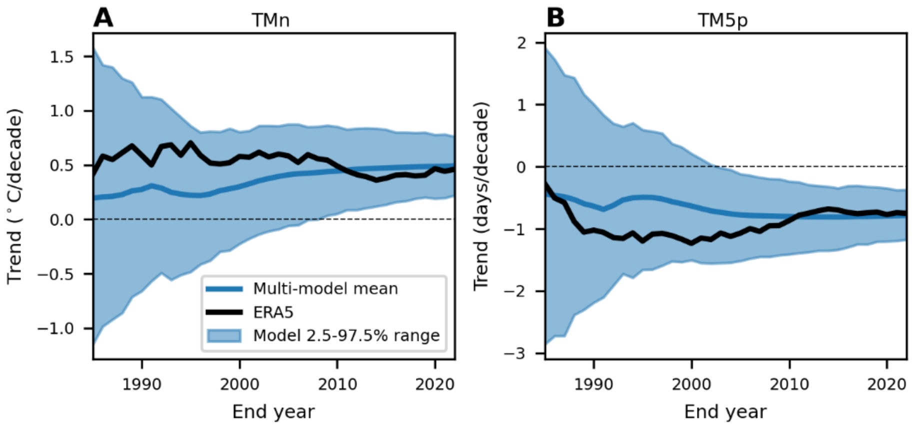

Fig. S3. Trends as a function of end year in observations and models. The magnitude of the trend starting in 1971 as a function of end year in ERA5 (black) for TMn (A) and TM5p (B). The blue shading shows that 2.5-97.5 percentile range of trends from the models and the blue line shows the trend for the multi-model mean.

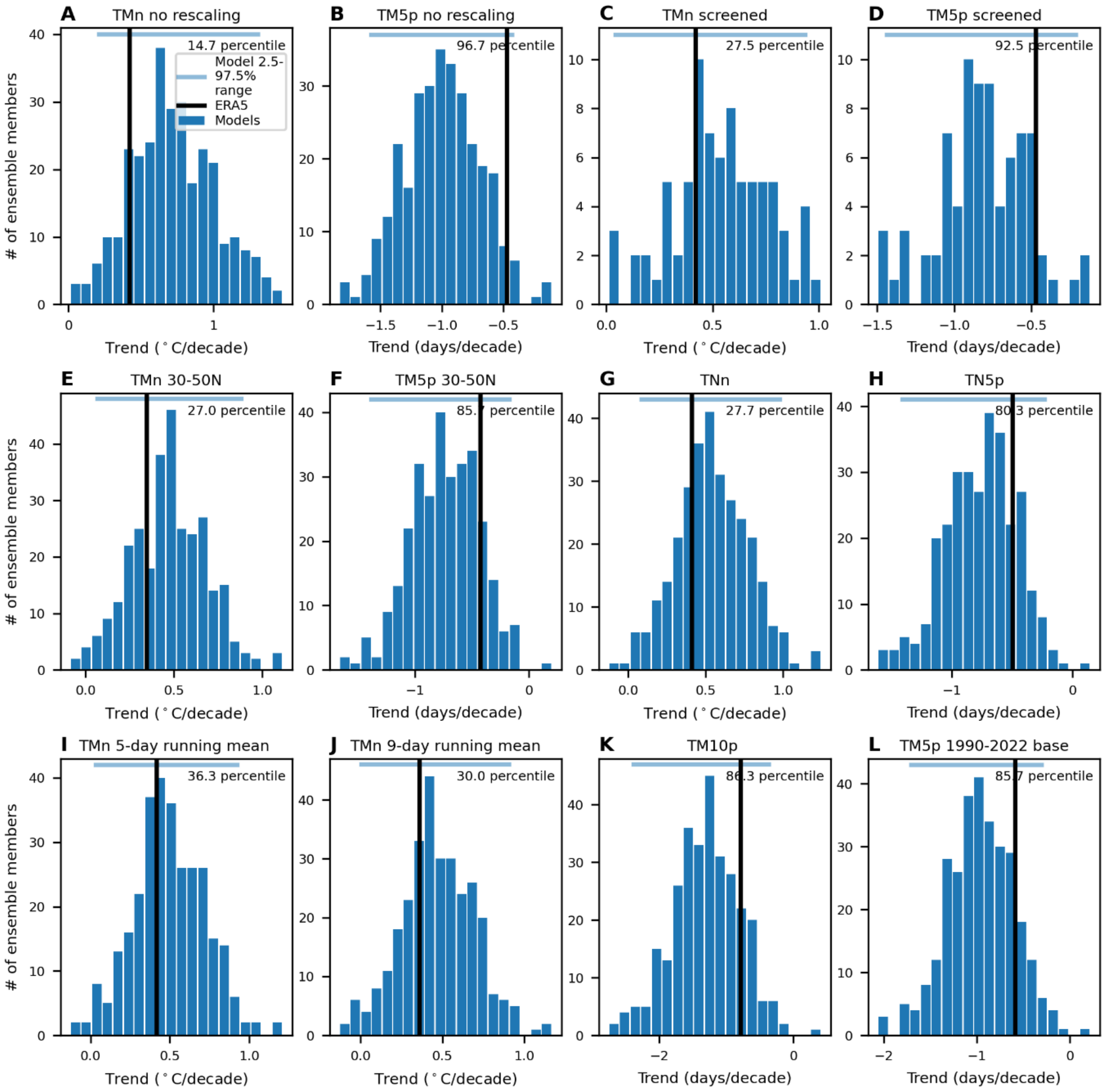

Fig. S4. Comparison of trends between models and observations for different choices made in the analysis. **A-H** As in Fig 2C,D, but without rescaling based on global mean temperatures (A, B), selecting models which closely match observed trends in global mean temperature(C, D), averaging over 30-50°N (E, F), and using daily minimum temperature for the metrics (G, H) **I**, **J** As in Fig 2C, but using the minimum temperature after a 5-day (I) and 9-day (J) running mean is applied. **K**, **L** As in Fig 2D, but using 10th percentile for the threshold (K), and using the 1990-2022 base period (L).

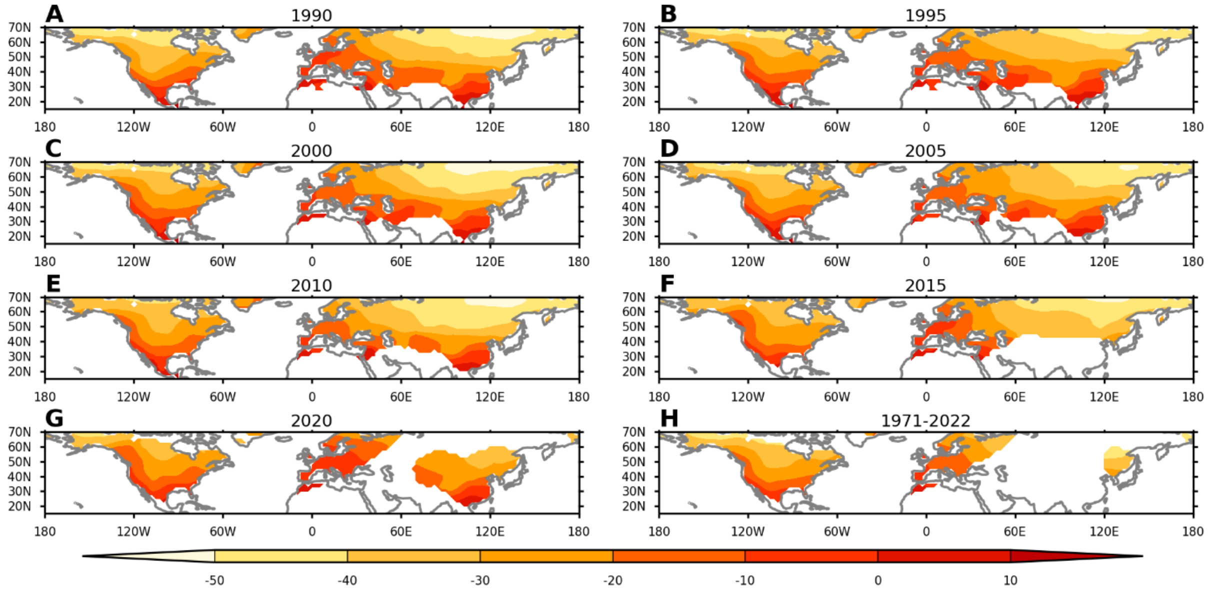

Fig. S5. Spatial coverage of the GHCNDEX dataset for TNn. A-G TNn (°C) from GHCNDEC plotted for different years from 1990 to 2020 in 5-year intervals (A-G), indicating the spatial coverage of the dataset. **H** Average of TNn over 1971-2022 for grid points with data over the entire period, indicating the mask that is used.

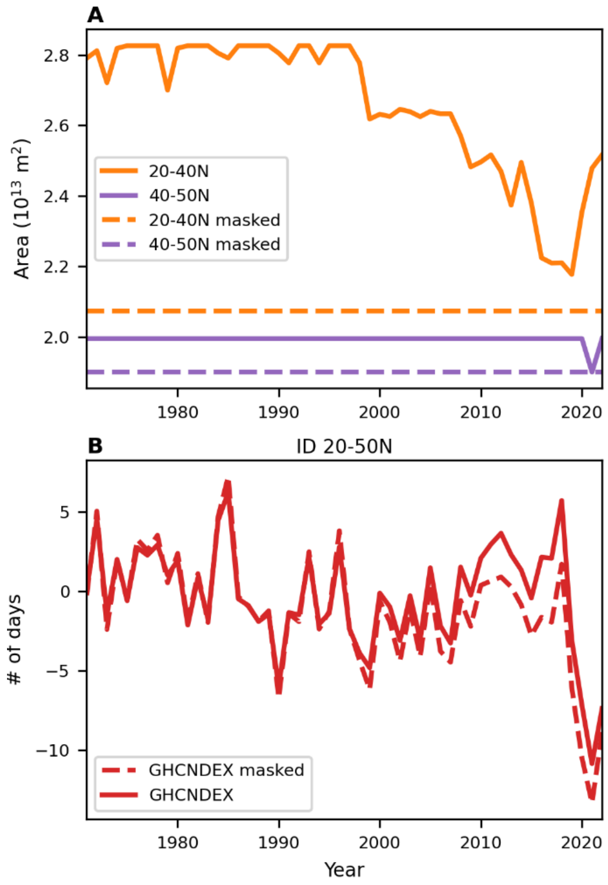

Fig. S6. Correcting for artefacts of changing spatial coverage of data for Icing Days. As in Fig 3A,B but for Icing Days(ID).

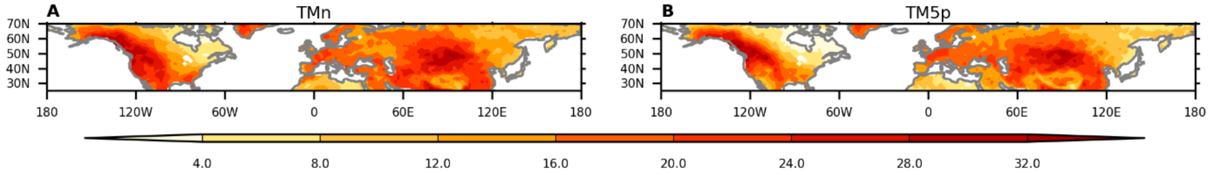

Fig. S7. Probability of increases in cold extremes over 1990-2022 in modelled trends. The percentage of ensemble members with TMn less than 0 (**A**) and with TM5p trends greater than 0 (**B**) over the 1990-2022 period.

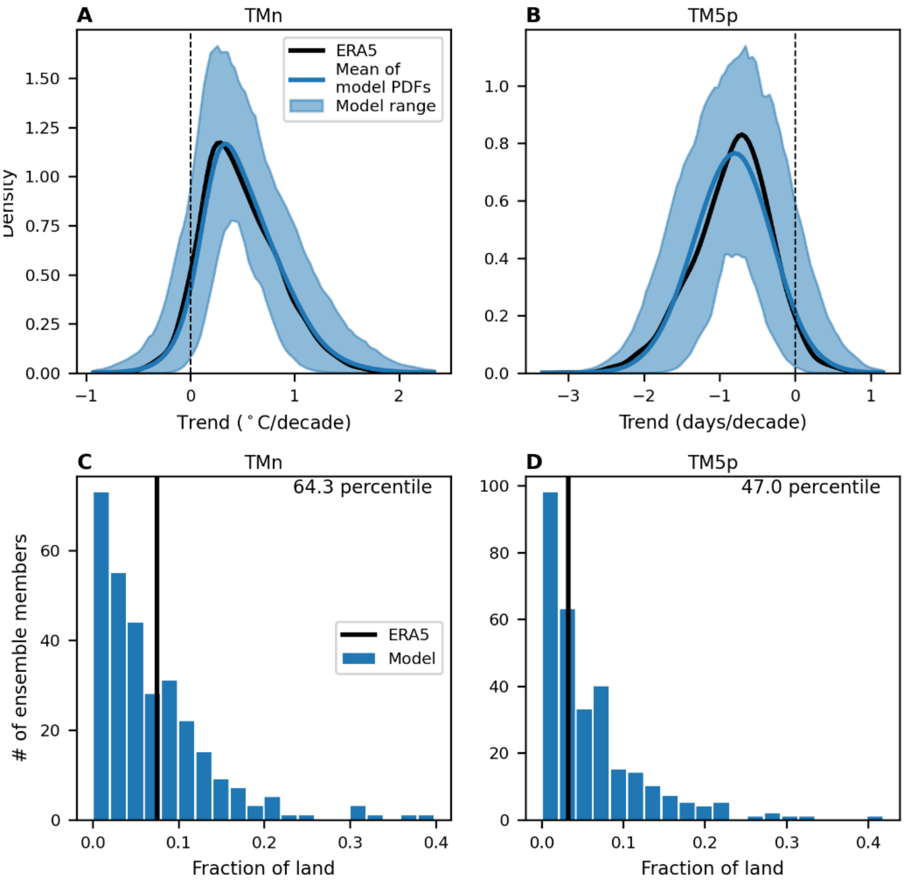

Fig. S8. Spatial distribution of cold extreme trends in observations and models. As in Fig 5, but for 1971-2022 trends.

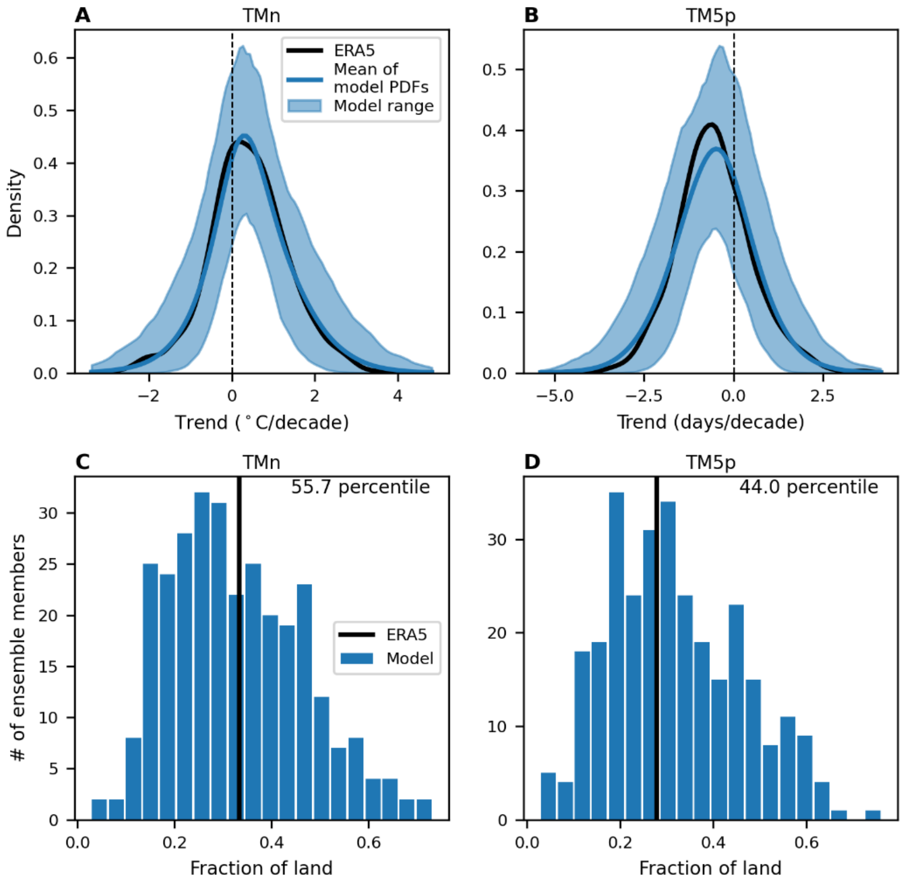

Fig. S9. Spatial distribution of cold extreme trends in observations and models. As in Fig 5, but for 2000-2022 trends.

Model	# of ensemble members	Scenario used for 2015-2022
ACCESS-ESM1-5	40	SSP3-7.0
CanESM5	50	SSP2-4.5
CESM2	50	SSP3-7.0
EC-Earth3	50	SSP5-8.5
GFDL-SPEAR-MED	30	SSP5-8.5
MIROC6	50	SSP5-8.5
MPI-ESM1-2-LR	30	SSP5-8.5

Table S1. Overview of the models used in this study. A list of the seven models used in this study, the number of ensemble members, and the scenario used for the extension from 2015-2022.

Region	1990-2022 TMn (%)	1990 -2022 TM5p (%)	2000-2022 TMn (%)	2000-2022 TM5p (%)
Midlatitude land	75	84	46	53
CEUS	21	23	14	13
SSNC	19	22	10	12
NEUR	35	28	19	18
SEUR	24	20	12	11

Table S2. The precentage of ensemble members with statitically significant decreases in cold extremes over selected regions. Trends are calculated for both TMn an TM5p and over both 1990-2022 an 2000-2022. Statistical significance is determined using a Student's t-test with a p=0.05 threshold. In addition to the midlaitude (30° to 60°) land average, we also use the regions defined in (*42*): Central and Eastern United States (CEUS; 30° to 50°N, -106° to -91°E), Southern Siberia and Northern China (SSNC; 40° to 60°N, 80° to 120°W), Northern Europe (NEUR;50° to 65°N, 0° to 45°W), and Southern Europe (SEUR;40°to50°N, 0° to 45°W).
